# Studies of Laboulbeniales on *Myrmica* ants (III): myrmecophilous arthropods as alternative hosts of *Rickia wasmannii*

**DOI:** 10.1051/parasite/2016060

**Published:** 2016-11-16

**Authors:** Walter P. Pfliegler, Ferenc Báthori, Danny Haelewaters, András Tartally

**Affiliations:** 1 Department of Biotechnology and Microbiology, University of Debrecen Debrecen Hungary; 2 Department of Evolutionary Zoology and Human Biology, University of Debrecen Debrecen Hungary; 3 Department of Organismic and Evolutionary Biology, Harvard University Cambridge Massachusetts USA

**Keywords:** Acari, Ecological specificity, Formicidae, Fungal parasite, *Microdon myrmicae*, Parasitism

## Abstract

Myrmecophilous arthropods and their manifold relations to host ants are interesting from an evolutionary perspective. *Rickia wasmannii* is an ectoparasitic fungus belonging to the Laboulbeniales order. Here, we show that inquiline mites can become infected by *R. wasmannii*, which was thought to be restricted to the genus *Myrmica* (Hymenoptera: Formicidae). This is the first report of *R. wasmannii* from an alternative host in another subphylum (Chelicerata). We also found immature fruiting bodies on a larva of *Microdon myrmicae* (Diptera: Syrphidae), which represents the first report of any *Rickia* species on flies. This fungus is capable of infecting alternative, unrelated host species as they co-occur in the ant nest “microhabitat”. These observations provide direct evidence for ecological specificity in Laboulbeniales. The presence of *R. wasmannii* on inquilines in *Myrmica* ant nests suggests that the parasite may have adapted to the ant nest environment and is less dependent on acquiring specific nutrients from the hosts. However, the alternative cannot be excluded; these infections might also represent chance events if the fungus is incapable of fulfilling its life cycle.

## Abstract (fr)

Les arthropodes myrmécophiles et leurs relations multiples avec leurs fourmis hôtes sont intéressants d’un point de vue évolutif. *Rickia wasmannii* est un champignon ectoparasite appartenant à l’ordre Laboulbeniales. Ici, nous montrons que les acariens inquilins peuvent être infectés par *R. wasmannii*, que l’on croyait limité au genre *Myrmica* (Hymenoptera : Formicidae). Ceci est le premier signalement de *R. wasmannii* chez un hôte différent dans un embranchement différent (Chelicerata). Nous avons également trouvé des fructifications immatures sur une larve de *Microdon myrmicae* (Diptera : Syrphidae), ce qui représente le premier signalement d’une espèce de *Rickia* sur une mouche. Ce champignon est donc capable d’infester des espèces hôtes non apparentées qui sont présentes dans le « microhabitat » des nids de fourmis. Ces observations fournissent la preuve directe de la spécificité écologique des Laboulbeniales. La présence de *R. wasmannii* sur des inquilins dans les nids des fourmis de genre *Myrmica* suggère que le parasite peut s’être adapté à l’environnement des nids de fourmis et est moins dépendant de l’acquisition de nutriments spécifiques des hôtes. Cependant, l’alternative ne peut être exclue : ces infections peuvent également représenter des événements accidentels si le champignon est incapable de finir son cycle de vie.

## Introduction

Social symbionts, referred to as “inquilines”, are those insects and other arthropods that live in the nest of their ant hosts (Hymenoptera: Formicidae) and have some obligatory, symbiotic relationship with them. These symbionts can be parasites, commensals, or mutualists. Relationships between ants and their diverse inquiline (= myrmecophilous) arthropod species (mites, isopods, springtails, bristletails, crickets, flies, butterflies, beetles, etc. [18]) are shaped by multiple factors. Inquilines are greeted with a stable microclimate, abundant food, protection from predators, and protection from most microbial pathogens by a “social immunity” in the ant nest “microhabitat” [14, 18, 21, 24, 34, 36, 37]. This social immunity generally results in reduced virulence. As a result, parasites of insect societies are thought to be less damaging to their hosts than those associated with non-social hosts [19]. Ant colonies, on the other hand, can harbor a diversity of highly specialized parasitic microorganisms [18, 45] and the possibility of myrmecophilous arthropods acquiring some of these associates cannot be excluded.

### Laboulbeniales biotrophic parasites

The Laboulbeniales (Fungi: Ascomycota: Laboulbeniomycetes) represent a highly diversified but understudied example of fungal biotrophs that live attached to the exterior of their arthropod hosts. Hosts are members of three subphyla in the Arthropoda: Chelicerata, Myriapoda, and Hexapoda. Six species of this order are associated with ants: *Dimorphomyces formicicola* (Speg.) I.I. Tav., *Laboulbenia camponoti* S.W.T. Batra, *L. ecitonis* G. Blum, *L. formicarum* Thaxt., *Rickia lenoirii* Santam., and *R. wasmannii* Cavara [12, 13, 16, 17, 34].

Host shifts are probably an important driving force of speciation among Laboulbeniales fungi [11], as certain morphologically similar species are associated with phylogenetically unrelated hosts. For example, *Laboulbenia davidsonii* W. Rossi was described from cicindeline hosts (Coleoptera: Carabidae: Cicindelinae), although it is obviously related to a group of species parasitic on *Galerita* spp. (Coleoptera: Carabidae: Harpalinae) [30]. In addition, *L. littoralis* De Kesel & Haelew. and *L. slackensis* Cépède & F. Picard are sister taxa that also occur on two unrelated beetle hosts, *Cafius xantholoma* (Gravenhorst, 1806) (Coleoptera: Staphylinidae: Staphylininae) and *Pogonus chalceus* (Marsham, 1802) (Coleoptera: Carabidae: Trechinae), respectively. These hosts, however, are both halobiont, salt marsh-inhabiting species and occur in close proximity to seaweed and plant debris. Morphological and ecological evidence supported that a host shift between these unrelated but co-occurring hosts had happened, leading to reproductive isolation of populations (on these different hosts), changes in morphology, and speciation [11].

Plurivory of Laboulbeniales is an interesting phenomenon. First, most Laboulbeniales exhibit moderate to high host specificity. Often there is a one-to-one relationship between parasite and host. Thus, explaining how and why certain Laboulbeniales species have multiple hosts is difficult. Second, plurivory could ultimately lead to (ecological) speciation by reproductive isolation, since the different populations may be using different nutritional resources and environments. It has been suggested that specific nutrients of co-habiting hosts (or, alternatively, nutrients available from the hosts’ environment) may be far more important for Laboulbeniales species associated with multiple hosts than the identity of the insect hosts [3, 11, 37]. The best-known example of a Laboulbeniales species with multiple diverse host groups is *L. ecitonis*, reported in Brazil [7], Costa Rica [27], Ecuador [29], and Panama (Haelewaters, unpublished data). This fungus is known from *Eciton* Latreille, 1804 ants (Ecitoninae), *Sternocoelopsis auricomus* Reichensperger, 1923 (Coleoptera: Histeridae), *Ecitophya* spp. (Coleoptera: Staphylinidae), and uropodid mites (Acari: Mesostigmata: Uropodidae). These beetle and mite species are all associated with the *Eciton* ants.

### The genus *Rickia*

Two of the six Laboulbeniales species associated with ants belong to the genus *Rickia* Cavara. The most widespread species of the two is *R. wasmannii*, with reports from 17 European countries; it is found on 9 species in the genus *Myrmica* Latreille, 1804 [12, 16]. The second species, *R. lenoirii*, is known from *Messor wasmanni* Krausse, 1910 and *M. structor* (Latreille, 1798) in France, Greece, Hungary, and Romania [2, 34].

The genus *Rickia* includes many more species (a total of 161) [35] and is unusual among Laboulbeniales for several reasons. Morphologically, its receptacle is multiseriate (mostly triseriate) and one cell layer thick. Its host distribution is very wide, encompassing three subphyla: Chelicerata (mites), Myriapoda [millipedes (Diplopoda)], and Hexapoda [ants (Hymenoptera: Formicidae), cockroaches (Blattodea), mole crickets (Orthoptera), and various beetle families (Coleoptera)] [39, 44]. *Rickia* species also differ largely in size. The largest species was only recently described: *R. gigas* Santam et al., measuring up to 2.2 mm in total length. This is among the largest species in the order Laboulbeniales [32, 35]. Among the smallest *Rickia* species, most of them are “acarophilous”, that is, they occur on mites. Examples are *R. anomala* (48–56 μm), *R. depauperata* (35–40 μm), *R. excavata* (75–85 μm), and *R. parvula* (40 μm) [42]. However, other small *Rickia* species have also been described that are not associated with mites, such as *R. euxesti* (40–68 μm) on *Euxestus* spp. (Coleoptera, Cerylonidae), and *R. lenoirii* (45–67 μm) on *Messor* spp. (Hymenoptera, Formicidae) [34, 42].

In this study, we screened *Myrmica scabrinodis* Nylander, 1846 ants and associated myrmecophilous arthropods for possible infections with a well-known and easily recognized Laboulbeniales ectoparasite, *Rickia wasmannii* [8], in populations from Hungary. This fungus is only known to infect nine species of the genus *Myrmica* [17] and it is remarkable for its well-studied biology and effects on its hosts [1, 9, 16, 17, 23]. *Myrmica* ants are known to host several parasitic and inquiline arthropods in Central Europe: mites, larvae of *Microdon myrmicae* Schönrogge et al. 2002 (Diptera: Syrphidae) and *Maculinea* van Eecke, 1915 caterpillars (Lepidoptera: Lycaenidae) [45], all of which can co-occur within the same sites [40].

## Materials and methods

**Figure 1.**
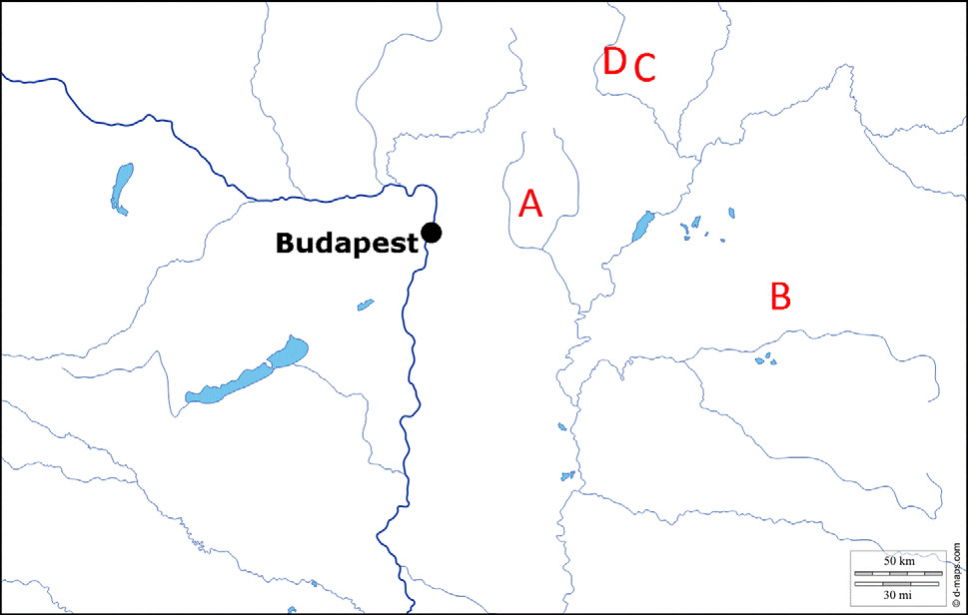
Collection sites in Hungary. A: Gyöngyös: Sár-hegy: Gyilkos-rét. B: Újléta. C: Rakaca: Meszes. D: Jósvafő: Tohonya-hát.

Ant colonies of *Myrmica scabrinodis* were collected in 2015 at the following sites in eastern and northern Hungary (Figure 1): 2 colonies from Gyöngyös: Sár-hegy: Gyilkos-rét (47°48′ N, 19°58′ E; 352 m a.s.l.); 3 colonies from Újléta (47°26′ N, 21°51′ E; 120 m a.s.l.); and 2 colonies from Rakaca: Meszes (48°27′ N, 20°47′ E; 165 m a.s.l.). We screened 60 workers for infection with *R. wasmannii* from each colony. Additionally, 1 syrphid larva (Diptera: Syrphidae) from Rakaca: Meszes (collected in 2012) and smaller collections of worker ants from Rakaca: Meszes (2014) and from Jósvafő: Tohonya-hát (48°29′ N, 20°32′ E; 268 m a.s.l) (2015) were screened for infection.

Ants and their associates were killed in ethanol and screened for fungal infection using a Leica MZ125 microscope at 10–160× magnification. Mites were mounted onto microscope slides in Heinz PVA Mounting Medium and screened at 10–100× magnification using a Carl Zeiss microscope with transmitted light.

Host species were determined according to [25] (ants) and [20] (mites). Fungal thalli were determined following [8, 12]. Immature thalli were determined based on the characteristically on the characteristically elongated basal cell of the thallus (= cell I).

## Results

**Figure 2.**
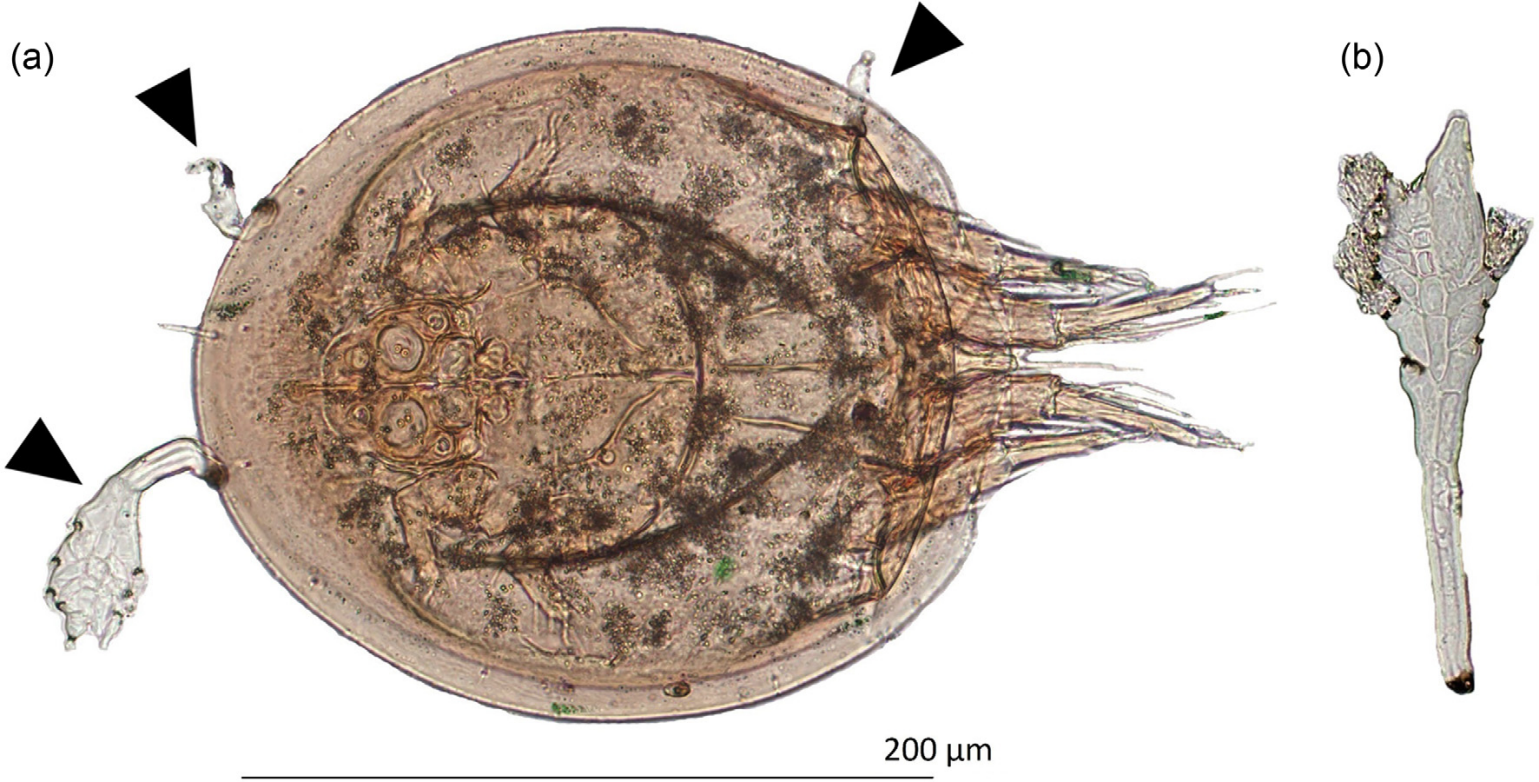
*Rickia wasmannii*. (a): Infected Acaridae deutonymph with three immature *R. wasmannii* thalli attached (marked). (b): Mature thallus from a *Myrmica scabrinodis* ant host. Scale bar = 200 μm.

Table 1 summarizes numbers of screened and infected ants and inquilines per *M. scabrinodis* colony. A total of 426 *M. scabrinodis* workers were collected and screened for Laboulbeniales. Four hundred twenty workers were infected with *R. wasmannii* (= 98.6%). In the sampled colonies, 62 mite specimens were found belonging to four families: Acaridae (*n* = 40), Histiostomatidae (*n* = 18), Neopygmephoridae (*n* = 1), and Scutacaridae (*n* = 1). The vast majority were phoretic deutonymphs of the Astigmatina “cohort”, which include the Acaridae and Histiostomatidae families. Altogether, 6 infected deutonymphs in the Acaridae family from a single colony in Gyöngyös: Gyilkos-rét were found (= 9.7% of all screened mites). In this colony, 33% of the Acaridae deutonymphs were infected, but none of the Histiostomatidae deutonymphs. All infected specimens bore 1 to 3 immature thalli. An example of an infected mite is shown in Figure 2a, with a mature thallus isolated from a *M. scabrinodis* worker for comparison (Fig. 2b). This is the first non-ant host record for *R. wasmannii*.

**Table 1.** Ants and ant colonies collected in Hungary, in the period 2012–2015, with indication of number of screened and infected ants and inquilines.

*M. scabrinodis* colony	*N* screened ants	*N* infected ants	Screened inquilines	*N*	*N* infected inquilines
Gyilkos-rét_4	60	54	Acaridae	18	6
			Histiostomatidae	12	
Gyilkos-rét_5	60	60	Acaridae	2	
			Histiostomatidae	2	
			Scutacaridae	2	
Újléta_2	60	60	Acaridae	4	
			Histiostomatidae	1	
Újléta_3	60	59	Acaridae	8	
Újléta_4	60	60	Acaridae	1	
Rakaca_3	60	60	Acaridae	6	
Rakaca_5	60	60	Histiostomatidae	1	
Rakaca_2014	3	3	Acaridae	1	
			Histiostomatidae	2	
Jósvafő_1	3	3	Neopygmephoridae	1	
			Scutacaridae	1	
Rakaca_2012	N/A	N/A	*Microdon* larva	1	1

Furthermore, two immature *Rickia* thalli are reported on the anterior horn of a *Microdon myrmicae* larva from a colony collected in Rakaca. This represents the first report of any *Rickia* species on Diptera.

## Discussion

The nature of the relationships between *R. wasmannii* and its newly recorded hosts pose several questions and imply parallels with other host-parasite relations within the Laboulbeniales order. Species of Laboulbeniales associated with mites are frequently found on the mites’ various host beetles as well [38, 42]. However, in many cases the parasite has only been recorded from the mite but not on its host insect [33, 38, 42]. Phoretic states of *Pyxidiophora* Bref. & Tavel (Pyxidiophorales, sister order of Laboulbeniales) are also relatively frequently reported on beetle-associated phoretic mites [4–6].

Of all *Rickia* species, 59 have been described from mites [22, 34, 41, 42]. Many of these are found exclusively on insect-associated mites (mostly those associated with Coleoptera) but not on the insects [33, 41, 42]. For example, three species of *Rickia* from Poland were described [22] on myrmecophilous mites belonging to different families of the order Mesostigmata from nests of *Lasius* spp. Neither of these *Rickia* species was found on the ants. Upon the discovery of *R. lenoirii* from *Messor* ants, its similarity to these extremely small mite-associated species was noted, suggesting that *R. lenoirii* may have evolved after a host shift from mites to ants [34]. Also in the case of *R. euxesti*, a species occurring on Cerylonidae, host shifts from associated mites to the beetle host could have happened [*sensu* 38, 41]. Another *Rickia* species, *R. kistneri*, was found on >50% of the *Mimaenictus wilsoni* Kistner & Jacobson, 1975 specimens (Coleoptera: Staphylinidae) [29]. These myrmecophilous beetles were collected together with >100 *Aenictus laeviceps* ants in the same emigrating column. However, none of the ants were infected [29]. Some species of the genus *Rickia* reported from ant species and/or their inquilines are listed in Table 2.

**Table 2.** *Rickia* parasitizing ants and/or associated (myrmecophilous) arthropods, with indication of the currently known distribution.

*Rickia* species	Ant host(s)	Myrmecophilous mite host(s)	Myrmecophilous beetle host(s)	Myrmecophilous fly host	Known distribution	Reference(s)
*Rickia depauperata* Thaxt.		Mesostigmata: Celaenopsidae (*Celaenopsis* sp.)			Haiti	[42]
*Rickia excavata* Thaxt.		Mesostigmata: Celaenopsidae (*Celaenopsis* sp.)			Trinidad	[42]
*Rickia georgii* T. Majewski		Mesostigmata: Dermanyssidae (*Hypoaspis cuneifer*)			Poland	[22]
*Rickia kistneri* W. Rossi			Staphylinidae (*Mimaenictus wilsoni*)		Peninsular Malaysia	[29]
*Rickia lenoirii* Santam.	*Messor* spp.				France, Greece, Hungary, Romania	[1, 34]
*Rickia macrochelis* Thaxt.		Mesostigmata: Macrochelidae (*Macrocheles* sp.)			Indonesia (Sumatra)	[42]
*Rickia nigriceps* Thaxt.		Mesostigmata: Euzerconidae (*Euzercon* sp.)			Solomon Islands	[42]
*Rickia pachylaelapis* T. Majewski		Mesostigmata: Pachylaelapidae (*Sphaerolaelaps holothyroides*)			Poland	[22]
*Rickia parvula* Thaxt.		Mesostigmata: Celaenopsidae (*Celaenopsis* sp.), Euzerconidae (?*Euzercon* sp.)			Trinidad	[42]
*Rickia stellata* T. Majewski		Mesostigmata: Celaenopsidae (*Celaenopsis* spp., *Pleuronectocelaeno* spp.)			Poland	[22]
*Rickia wasmannii* Cavara	*Myrmica* spp.				Europe	[12, 16]
	*Myrmica scabrinodis*	Astigmatina: Acaridae		Syrphidae (*Microdon myrmicae*)	Hungary	Present paper

### Ecological dead-ends?

Our report of *Rickia* thalli on a single *Microdon myrmicae* larva represents the first report of any species of Laboulbeniales on Syrphidae. The extremely low parasite load on the relatively large *M. myrmicae* larva (two immature thalli) indicates that this infection may have been accidental. Laboulbeniales occur practically exclusively on adults. Infections of eggs, larvae, pupae, or nymphs are extremely rare, but have been reported in cockroaches, termites, beetles, and ants [3, 28, 31]. In cockroaches, *Herpomyces* spp. are found on both the adults and co-habiting nymphs, although upon ecdysis, the infection is lost [28]. As to beetles, a single immature specimen of *Systena s-littera* (Linnaeus, 1758) from Brazil was reported to carry *Laboulbenia systenae* Speg. [31].

The infected mites and the single *M. myrmicae* larva bore only immature thalli. We cannot exclude the possibility that using alternative hosts may be deleterious for the fungus. Alternative hosts thus may provide only suboptimal conditions for the fungus. Furthermore, mite deutonymphs and fly larvae both undergo ecdysis and thus Laboulbeniales thalli will be lost [*sensu* 28]. In these cases, the accidental colonization of new hosts may be dead-ends for *R. wasmannii*. Further studies on the highly diverse arthropod community of *Myrmica* nests [45] could identify more hosts of *R. wasmannii* and help in answering questions about the life history strategies of this parasite.

### Microhabitats

*Rickia wasmannii* making use of multiple hosts in a different order (Diptera) and even a different subphylum (Chelicerata) as described here reminisces the tropical *L. ecitonis* on inquilines of *Eciton* ants [3, 7]. In this case, the ant colony itself (of which the individual members form a “living nest”) serves as a “microhabitat” where ascospores can be transmitted to unusual myrmecophilous hosts. Other examples of a microhabitat are saltmarshes, subterranean caves, and wet, decomposing logs [11, 26, 38]. Several complex associations between log-inhabiting arthropods, their associated mites, and *Rickia* (as well as *Dimorphomyces*) species were described from Queensland, Australia [38]. *Rickia berlesiana* was found to be the most plurivorous one, recorded from several species of Fedrizziidae (Acari: Mesostigmata) as well as three species of Passalidae beetles hosting the mites [38]. These results indicate the use of multiple alternative hosts in two subphyla.

The presence of *R. wasmannii* on inquilines in *Myrmica* ant nests suggests that *R. wasmanni* may have adapted to the ant nest environment and is less dependent on acquiring specific nutrients from the hosts. In other words, ecological specificity is more important than host specificity. Tragust et al. [43] have shown that *R. wasmannii* has a non-penetrating hoof-like foot structure for attachment to the host. The fact that this species does not penetrate its host calls for another mode for nutrition. If *R. wasmannii* only needs the host for attachment to the cuticle, it could indeed be that nutrition happens at the cuticle or through the environment. This may explain why *R. wasmannii* does not need to be host specific because of restricted nutritional needs.

### Ecological specificity

The “easiness” of using non-ant hosts is particularly compelling when the apparent narrow host specificity of *R. wasmannii* is taken into account. Haelewaters et al. [16], for example, found no sign of transmission between infected *Myrmica* spp. and ants of other genera sharing the same narrow geographic area. The key factor enabling the usage of non-ant hosts may be the microhabitat, provided by the nest of the *Myrmica* ants: apparently, the fungus exhibits low host specificity, but only inside the ant nest microhabitat. Our records thus represent the third type of specificity alongside the well-known host specificity [10] and position specificity [15] in the order Laboulbeniales: ecological specificity [11].

Based on our observations, we do not know with certainty whether infection on inquilines in nests of *M. scabrinodis* is truly due to the fact that they represent alternative hosts (or even stable hosts shift events) for the fungus, or whether infection on inquilines represents chance events. However, the occurrence of infection on associated myrmecophiles may, over evolutionary time, lead to the use of myrmecophiles as alternative hosts for the fungus and, because of micro-evolutionary changes and reproductive isolation, potentially even to speciation.
